# Evaluation of early metabolic changes following vorasidenib using FET PET in patients with *IDH*-mutant gliomas

**DOI:** 10.1093/noajnl/vdae210

**Published:** 2024-11-29

**Authors:** Norbert Galldiks, Jan-Michael Werner, Isabelle Stetter, Hannah C Puhr, Thomas S Nakuz, Gabriele Stoffels, Nathalie L Albert, Karl-Josef Langen, Philipp Lohmann, Matthias Preusser

**Affiliations:** Center of Integrated Oncology Aachen Bonn Cologne Duesseldorf (CIO ABCD), Cologne, Germany; Institute of Neuroscience and Medicine (INM-3, INM-4), Research Center Juelich, Juelich, Germany; Department of Neurology, Faculty of Medicine and University Hospital Cologne, Cologne, Germany; Division of Oncology, Department of Medicine I, Medical University of Vienna, Vienna, Austria; Department of Neurology, Faculty of Medicine and University Hospital Cologne, Cologne, Germany; Department of Neurology, Faculty of Medicine and University Hospital Cologne, Cologne, Germany; Division of Oncology, Department of Medicine I, Medical University of Vienna, Vienna, Austria; Division of Nuclear Medicine, Department of Biomedical Imaging and Image-Guided Therapy, Medical University of Vienna, Vienna, Austria; Institute of Neuroscience and Medicine (INM-3, INM-4), Research Center Juelich, Juelich, Germany; Department of Nuclear Medicine, LMU University Hospital, Munich, Germany; Department of Nuclear Medicine, University Hospital RWTH Aachen, Aachen, Germany; Center of Integrated Oncology Aachen Bonn Cologne Duesseldorf (CIO ABCD), Cologne, Germany; Institute of Neuroscience and Medicine (INM-3, INM-4), Research Center Juelich, Juelich, Germany; Department of Nuclear Medicine, University Hospital RWTH Aachen, Aachen, Germany; Institute of Neuroscience and Medicine (INM-3, INM-4), Research Center Juelich, Juelich, Germany; Division of Oncology, Department of Medicine I, Medical University of Vienna, Vienna, Austria

## Abstract

The phase-3 INDIGO trial demonstrated that the isocitrate dehydrogenase (*IDH*) inhibitor vorasidenib significantly prolonged progression-free survival and delayed intervention in patients with CNS WHO grade 2 gliomas. However, conventional MRI showed limited response, with only 11% of patients having objective responses. Studies suggest that serial PET imaging with radiolabeled amino acids, such as *O* -(2-[^18^ F]-fluoroethyl)-L-tyrosine (FET) PET, may provide earlier and more informative assessments of treatment response than MRI. In an initial experience with FET PET, 3 out of 5 patients showed metabolic response to vorasidenib. This highlights FET PET’s potential to guide decision-making, though further trials are needed to confirm outcome benefits.

The phase-3 INDIGO trial suggested that the *IDH* inhibitor vorasidenib significantly prolonged progression-free survival (PFS) and the time to the next intervention in patients with residual or recurrent CNS WHO grade 2 gliomas.^1^ In this trial, MRI-based PFS and response rates were evaluated using the RANO criteria for low-grade gliomas on the basis of changes in T2/fluid attenuated inversion recovery (FLAIR) hyperintensity. Of note, most patients (83%) had unchanged MRI findings after treatment initiation, and objective responses were observed in only 11% of patients.^1^ Thus, an earlier assessment of response following *IDH* inhibitors would be of considerable clinical value for patient counseling.^2,3^

A growing body of literature suggests that serial PET imaging using radiolabeled amino acids for evaluating response compared to anatomical MRI provides valuable additional clinical information for decision-making. In particular, it has been reported that metabolic responders on amino acid PET to local (eg, radiotherapy with concomitant temozolomide chemotherapy) and systemic treatment options (eg, alkylating and antiangiogenic agents, targeted therapies) predicted a significantly longer survival than metabolic nonresponders and MRI responders.^3,4^

Here, we report initial experience with serial PET imaging using the radiolabeled amino acid *O*-(2-[^18^F]-fluoroethyl)-L-tyrosine (FET) for the assessment of response to vorasidenib in patients with nonenhancing CNS WHO grade 2 or 3 gliomas treated according to the INDIGO trial. Notably, all patients were treated off-study and received vorasidenib after enrollment in a Compassionate Use-Program, launched in 2024 in Europe by the distributing company. This expanded access program also allows the inclusion of patients with CNS WHO grade 3 gliomas. The neuroimaging evaluation presented here has been approved by the ethics committees of our institutions (EK 2086/2023; EK 096/18). All patients received a daily dose of 40 mg vorasidenib, and no grade 3 or 4 adverse events according to the Common Terminology Criteria for Adverse Events (CTCAE) were reported. None of the patients had previously undergone radiotherapy or systemic anticancer therapy. In 3 of 5 patients (Table 1), the metabolic activity decreased as assessed by a decline of mean tumor-to-brain-ratios^5^ early after vorasidenib initiation (range, 5.4-8.0 weeks) and indicated therefore metabolic response, whereas the hyperintensity on the T2/FLAIR MRI sequence remained unchanged (Figure 1). Moreover, in these 3 patients, the criteria for PET-based response *Partial Response* according to the recently defined PET RANO 1.0 were fulfilled.^6^ In one patient (#3 in Table 1) achieving PET-based *Stable Disease* following vorasidenib, PET-based *Progressive Disease* had been documented prior to initiation of vorasidenib.

**Table 1.** Overview of Patients’ Characteristics and Imaging Parameters

Pat. #	Sex, age	Tumor type	CNS WHO grade	No. of previous surgeries for glioma	Interventions/pretreatment before vorasidenib	Time between baseline and follow-up FET PET (weeks)	TBR_max/mean_ at baseline	TBR_max/mean_ at follow-up	MTV at baseline (mL)	MTV at follow-up (mL)	T2/FLAIR signal between baseline and follow-up	Response according to the PET RANO 1.0 criteria
1	M, 33	ODG	3	0	B, PR	6.1	3.5/2.1	2.9/1.9	4.4	2.7	Unchanged	PET-PR
2	M, 34	A	2	0	B	8.0	2.8/2.0	2.0/1.7	1.6	0.3	Unchanged	PET-PR
3	M, 41	A	2	0	B	8.7	2.2/1.8	2.1/1.8	6.1	8.0	Unchanged	PET-SD
4	M, 42	ODG	2	0	PR	5.4	1.8/1.7	NMD	0.6	NMD	Unchanged	PET-PR
5	M, 36	ODG	3	1 (CR)	PR	5.3	No uptake^a^	No uptake^a^	No uptake^a^	No uptake^a^	Unchanged	PET-SD

^a^No measurable disease according to the PET RANO 1.0 criteria.

Abbreviations: A, astrocytoma; B, stereotactic biopsy; CR, complete resection; M, male; MTV, metabolic tumor volume; NMD, nonmeasurable disease (ie, visible lesion with an intensity value below a maximum tumor-to-brain ratio of 1.6 or an amino acid PET volume < 0.5 mL); ODG, oligodendroglioma; PET-PR, *partial response*; PET-SD, *stable disease*; PR, partial resection; TBR_max/mean_, maximum and mean tumor-to-brain ratios on FET PET calculated according to the current guideline.^5^

**Figure 1.**
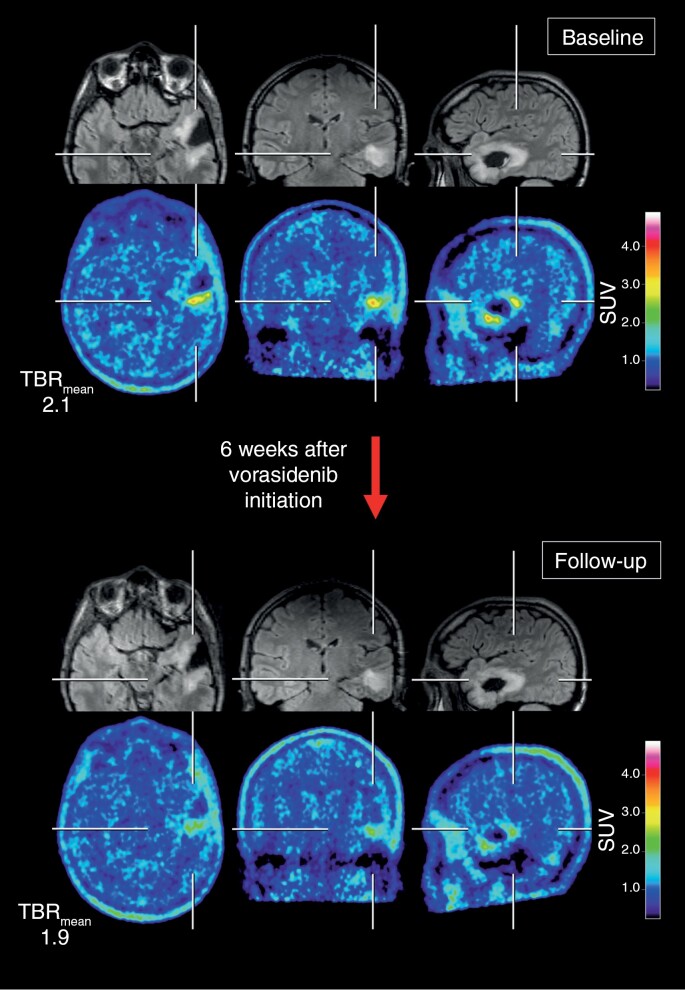
FLAIR MR and FET PET images of a 33-year-old male patient (patient #1) with a newly diagnosed and nonenhancing CNS WHO grade 3 oligodendroglioma at baseline (top rows) and at follow-up 6 weeks after vorasidenib initiation (bottom rows). After partial resection, the patient underwent vorasidenib therapy. In contrast to the unchanged hyperintense FLAIR signal, the metabolic activity decreased by 10% (ie, relative change in mean tumor-to-brain ratios from 2.1 to 1.9) indicating PET-based *Partial Response* according to the PET RANO 1.0 criteria. FET, *O*-(2-[^18^F]-fluoroethyl)-L-tyrosine.

In our view, this highlights the potential of FET PET to identify responders to vorasidenib considerably earlier after treatment initiation and may facilitate clinical decision-making. Nevertheless, whether a decline in metabolic activity on amino acid PET following vorasidenib as a surrogate for response is associated with an improved outcome, for example, prolonged PFS or OS, is yet unclear and warrants further investigation, preferably in clinical trials with predefined endpoints.

Despite the added clinical value of amino acid PET for evaluating response compared to anatomical MRI, the lack of uptake in a considerable fraction of patients with CNS WHO grade 2 or 3 gliomas (approximately 30%)^7^ may limit the usefulness of amino acid PET for assessing response to *IDH* inhibitors. This should be taken into account when planning future clinical trials. On the other hand, amino acid PET may help overcome shortcomings of other valuable methods to assess the effects of *IDH* inhibitors over time such as 2-hydroxyglutarate proton MR spectroscopy (2-HG MRS). In particular, amino acid PET is not affected by susceptibility artifacts related to local magnetic field distortions (eg, caused by mastoid air cells, bone, metallic material from surgery, paramagnetic blood products such as hemosiderin following surgery). Further obstacles of 2-HG MRS are the low signal intensity of 2-HG implying long measurement times, the limited spectral resolution in the field strengths of standard clinical scanners requiring high-field MR scanners, and a considerable rate of inaccuracies, for example, false-positively increased levels of 2-HG in *IDH*-wildtype glioblastomas.^8,9^
